# Carer subjective burden after first-episode psychosis: Types and predictors. A multilevel statistical approach

**DOI:** 10.1177/0020764020930041

**Published:** 2020-06-10

**Authors:** Shereen Charles, James B Kirkbride, Juliana Onwumere, Natasha Lyons, Lai Chu Man, Caroline Floyd, Kaja Widuch, Lucy Brown, Gareth James, Roya Afsharzadegan, Jonathan Souray, David Raune

**Affiliations:** 1 Division of Psychiatry, University College London, London, UK; 2 Harrow and Hillingdon Early Intervention in Psychosis Service, Central and North West London NHS Foundation Trust, London, UK; 3 Department of Psychology, Institute of Psychiatry, Psychology & Neuroscience, King’s College London, London, UK; 4 Bethlem Royal Hospital, South London and Maudsley NHS Foundation Trust, Beckenham, UK

**Keywords:** Carer burden, first-episode psychosis, illness beliefs, coping styles, psychosis, subjective burden

## Abstract

**Background:**

Carer burden at first-episode psychosis is common and adds to the multiple other psychiatric and psychological problems that beset new carers; yet, knowledge of the factors that predict carer burden is limited.

**Aim:**

This study sought to investigate the types and predictors of carer burden at first-episode psychosis in the largest, most ethnically diverse and comprehensively characterised sample to date.

**Method:**

This study involved a cross-sectional survey of carers of people with first-episode psychosis presenting to Harrow and Hillingdon Early Intervention in Psychosis service between 2011 and 2017. Carers completed self-report measures assessing their illness beliefs, coping styles and caregiving experiences (i.e. burden). Thirty carer and patient sociodemographic and clinical factors were also collected. Mixed effects linear regression modelling was conducted to account for clustering of carers by patient, with carer burden (and its 8 subtypes) investigated as dependent variables.

**Results:**

The sample included data on 254 carers (aged 18–74 years) and 198 patients (aged 14–36 years). Regression modelling identified 35 significant predictors of carer burden and its subtypes at first-episode psychosis. Higher total burden was independently predicted by perceiving greater negative consequences of the illness for the patient (B = .014, *p* < .001, 95% CI: [.010–.018]) and the carer (B = .008, *p* = .002, 95% CI: [.003–.013]), and engaging in avoidant-focussed coping (B = .010, *p* = .006, 95% CI: [.003–.016]). Lower burden was independently predicted by patients being in a relationship (B = −.075, *p* = .047, 95% CI: [−.149 to −.001]). Predictors of the eight burden subtypes (difficult behaviours, negative symptoms, stigma, problems with services, effects on family, dependency, loss and need to backup) are also included in the article.

**Conclusion:**

Findings can be used to inform the identification of carers ‘at-risk’ of experiencing burden and highlight potential targets for theraputic intervention to lower carer buden.

Informal caregivers play a vital role in the process of recovery and prevention of relapse following a first psychotic episode, through facilitating help and engagement with services (Alvarez-Jimenez et al., 2012; Fridgen et al., 2012; Revier et al., 2015). Although many report positive experiences, carers may also become particularly distressed by their role, and for this reason, reports of subjective burden are common (see Jansen et al., 2015 for a review).

Subjective burden (hereafter referred to as ‘burden’) refers to the degree of mental or psychological toll carers experience as a result of fulfilling their caregiving duties (Montgomery et al., 1985), and has existed as a clinically useful concept since the 1960s (Hoenig & Hamilton, 1966). Burden has been shown to serve as a marker for other psychological morbidity and adds to the multiplicity of psychiatric disorders that beset new carers such as anxiety, depressionand post-traumatic stress-disorder (e.g. Barton & Jackson, 2008; Hamaie et al., 2016; Onwumere et al., 2018; Raune & Kuipers, 2000; Raune et al., 2004; Sadath et al., 2017). Approximately one-third of carers looking after patients who are ‘at-risk’ for, or who have experienced their first psychotic episode will meet the criteria for a psychiatric disorder (Hamaie et al., 2016). This number has been shown to rise up to 57% in carers of individuals with chronic psychosis (Barrowclough & Parle, 1997). This is of considerable importance given that the majority of patients with psychosis reside with their families (50%–70%), particularly during the early stages of the illness (Addington et al., 2001). Hence, an understanding of which carers are most at-risk, and why, is crucial, especially considering a key element of early intervention involves reducing the likelihood of chronic distress for the whole family (Jansen et al., 2015).

When considering which carers are most at-risk, higher burden at first-episode psychosis has been reported in carers who are older (Addington et al., 2003; Boydell et al., 2014) and female (Möller-Leimkühler & Obermeier, 2008; Tennakoon et al., 2000), though findings in are inconsistent (e.g. Boydell et al., 2014; Möller-Leimkühler, 2005). Caring for a younger patient (Addington et al., 2003), with a diagnosis of schizophrenia (Boydell et al., 2014) and a younger illness onset age (Addington et al., 2003) has also been shown to predict higher levels of carer burden. A wider range of patient and carer characteristics that contribute to carer burden are yet to be determined.

In an attempt to explain why carer burden develops, there is growing evidence to support the role of illness beliefs and coping strategies as predictive factors, as captured by the stress-appraisal coping model (Lazarus & Folkman, 1984; Szmukler et al., 1996). However, research thus far has largely focussed on carers of individuals with chronic psychosis (e.g. Barrowclough et al., 2001; Fortune et al., 2005; Kuipers et al., 2007) in comparison to first-episode psychosis (Onwumere et al., 2008; Patel et al., 2014). Where first-episode psychosis is concerned, there is preliminary evidence to suggest that carers who have an external locus of control, and perceive a more chronic patient illness, report higher levels of burden (Onwumere et al., 2008; Patel et al., 2014). Higher levels of burden has also been identified in carers who anticipate greater negative consequences of the illness for themselves, and for the patient (Onwumere et al., 2008). Among the instruments used to assess illness beliefs, The Illness Perception Questionnaire (Barrowclough et al., 2001) is most commonly used. In 2005, it was expanded (Lobban et al., 2005) to incorporate several other key dimensions of experience. These dimensions include illness identity, causes of the illness, personal blame, treatment control and illness coherence, which, in the context of carer burden, have not yet been investigated.

Research on coping at first-episode psychosis is more established. Greater use of avoidant-focussed coping strategies has been shown to predict higher levels of carer burden more often (Cotton et al., 2013; Hinrichsen & Lieberman, 1999; Onwumere et al., 2011; Scazufca & Kuipers, 1999) than not (Gerson et al., 2011). Emotion-focussed and problem-focussed coping have also been linked to higher carer burden, though in the case of the latter, research is conflicting (Möller-Leimkühler, 2005; Tennakoon et al., 2000). Research has also assessed the relationship between the three coping strategies and various burden domains. Specifically, avoidant-focussed coping has been linked to higher carer burden in the domains of difficult behaviours, negative symptoms, stigma, problems with services, effects on the family, dependency, loss and perceiving a need to provide ‘backup support’ (Cotton et al., 2013). Problem-focussed coping has been linked to higher levels of carer burden with regard to the effect on family (Tennakoon et al., 2000). Emotion-focussed coping has similarly been linked to higher levels of familial burden, but also burden by dependency (Möller-Leimkühler, 2005; Tennakoon et al., 2000).

Although the aforementioned studies highlight some important predictors of carer burden at first-episode psychosis, many gaps in the literature remain. Findings pertaining to sample characteristics and coping styles are inconclusive, and there is a paucity of literature on illness beliefs. Existing studies on carer burden at first-episode psychosis have also been hampered by methodological limitations including small sample sizes and limited ethnic diversity, rendering results difficult to generalise beyond Caucasian females from an English-speaking background (Boydell et al., 2014; Cotton et al., 2013). Moreover, previous research has reported results on one carer only (Addington et al., 2003; Cotton et al., 2013; Hinrichsen & Lieberman, 1999; Möller-Leimkühler & Obermeier, 2008; Onwumere et al., 2008, 2011; Scazufca & Kuipers, 1999; Tennakoon et al., 2000), despite patients with psychosis often reporting having multiple carers (see Jansen et al., 2015 for a review). To address these gaps, the current study sought to establish the predictors of carer burden at first-episode psychosis. Where these variables are concerned, this study contains the largest, most ethnically diverse and comprehensively characterised sample to date.

## Hypotheses

Hypotheses were formed before data collection and where there was previous literature to draw upon, as detailed in Table 1. Where hypotheses have not been formed, we consider the research to be exploratory by nature.

**Table 1.** Hypotheses for this study.

No.	Hypothesis	Supporting literature
1	Carers who look after younger patients will report higher levels of total negative burden.	Addington et al. (2003)
2	Carers who look after a patient with a diagnosis of schizophrenia will report higher levels of total negative burden.	Boydell et al. (2014)
3	Carers who look after a patient with a younger illness onset age will report higher levels of total negative burden.	Addington et al. (2003)
4	Carers who perceive a more chronic illness timeline will report higher levels of total negative burden.	Onwumere et al. (2008)
5	Carers who perceive greater negative consequences of the illness for themselves and the patient will report higher levels of total negative burden.	Onwumere et al. (2008)
6	Carers who adopt more avoidant-focussed coping styles will report higher levels of total negative burden, and specifically, in the domains of difficult behaviours, negative symptoms, stigma, problems with services, effects on the family, dependency, loss and need to back up.	Hinrichsen and Lieberman (1999); Scazufca and Kuipers (1999); Onwumere et al. (2011) and Cotton et al. (2013)
7	Carers who adopt more emotion-focussed coping styles will report higher levels of total negative burden, and specifically in the domains of the effect on family, and dependency.	Tennakoon et al. (2000) and Möller-Leimkühler (2005)
8	Carers who adopt more problem-focussed coping styles will report higher levels of burden with regard to the effect on family.	Tennakoon et al. (2000)

## Method

### Design and sample

This was a cross-sectional study that utilised data from carers of people with first-episode psychosis presenting to the Harrow and Hillingdon Early Intervention in Psychosis Service between July 2011 and January 2017. The service is part of the National Health Service (NHS) and accepts individuals aged between 14 and 35 years who meet the criteria for a first-psychotic episode. All service users with an identifiable carer were eligible for inclusion in the study. Carers were described as those who assumed an unpaid caregiving role for an identified patient. Carer assessments were conducted face-to-face, via phone or email by graduate-level assistant psychologists following the patient’s referral to the service. In many instances, patients had more than one carer, so data were collected for each carer separately and were treated as independent entries. All carers included in this study gave written informed consent for publication.

### Measures

#### Predictor variables

##### Sample characteristics

In total, 18 carer demographic characteristics were collected including ethnicity, relationship status and weekly hours of face-to-face contact with the patient.

A total of 12 patient demographic and clinical characteristics were collected including diagnosis category, age at illness onset and duration of untreated psychosis.

##### Illness beliefs

Illness beliefs were assessed using the 147-item Illness Perception Questionnaire for Schizophrenia: Relatives Version (IPQS-RV; Lobban et al., 2005). Respondents rated their agreement with a series of statements on a 5-point Likert-type scale, ranging from 1 (*strongly disagree*) to 5 (*strongly agree*). The subscales used in this study were as follows: consequences of the illness (carer and patient subscales), control over the illness (carer and patient subscales), chronicity of the illness, blame (carer and patient subscales), treatment control (carer and patient subscales) and illness coherence. The IPQS-RV subscales used in this study have been shown to have good internal consistency and have been used in first-episode psychosis carer populations (e.g. Barrowclough et al., 2014; Lobban et al., 2005).

##### Coping styles

Coping strategies were assessed using the 30-item Coping Orientation to Problems Experienced (COPE) Inventory (Carver et al., 1989; Carver & Scheier, 1994). The COPE consists of 15 subscales which can be grouped to produce three broad styles of coping: avoidant-focussed coping (denial, focus on and venting of emotions, behavioural disengagement, alcohol-drug disengagement, mental disengagement); problem-focussed coping (active coping, planning, suppression of competing activities, restraint coping, seeking of instrumental social support) and emotion-focussed coping (seeking of emotional social support, positive reinterpretation and growth, acceptance, turning to religion, humour). Respondents indicated how often they engaged in a variety of practices on a 4-point Likert-type scale, ranging from 1 (*I have never done this*) to 4 (*I have done this a lot*). The COPE subscales have good internal consistency and have been used in first-episode carer populations (e.g. Baumstarck et al., 2017; Gerson et al., 2011; Raune et al., 2004).

#### Outcome variables

##### Burden

Carer burden was assessed using the 66-item Experience of Caregiving Inventory (ECI; Szmukler et al., 1996). The ECI was considered the most robust instrument, given the various validation limitations concerning alternative measures (see Reine et al., 2004 for a review). The ECI is one of few that has been validated using a factorial analysis and has been additionally assessed for external validity. Alternative measures of carer burden have also been criticised on the basis that they are not grounded in existing psychological theory. From this perspective, the ECI assesses caregiving appraisals within a stress-coping paradigm (Lazarus & Folkman, 1984). Respondents rated how frequently they thought about aspects of their caregiving role or specific problems over the previous month on a 5-point Likert-type scale, ranging from 0 (*never)* to 4 (*nearly always*). The following subscales were used in the present study: difficult behaviours, negative symptoms, stigma, problems with services, effects on family, dependency, loss and need to backup. Combining scores from each of the subscales produced a total negative burden score, which was the main outcome measure used within the present study. Negative total scores ranged from 0 to 208, with higher scores indicating greater levels of carer burden. As secondary outcomes, we also considered each of the eight ECI subscales separately. The ECI subscales used in this study have good to excellent internal consistency (α = .74–.91). The ECI also has strong face validity and has been extensively used in first-episode psychosis carer populations (e.g. Patterson et al., 2005; Szmukler et al., 1996; Tomlinson et al., 2013).

### Data analysis

Data were initially subject to descriptive statistics, using the SPSS statistical software, Version 24. Descriptive statistics were used to describe sample characteristics and to summarise carers’ ECI scores.

Inferential statistics were conducted in two phases for each of the eight ECI subscales and total negative burden. First, univariable mixed effects linear regressions (West et al., 2015) were conducted to investigate the relationship between potential predictor variables and burden outcomes. Mixed effects linear regressions were chosen to control for clustering within the data as many patients had more than one carer who participated in the study. The Bayesian Information Criterion (BIC) was obtained for each univariable regression model, where a lower BIC was indicative of a ‘better fit’. BIC values were subsequently ranked in ascending order. This would inform the order by which variables were removed from multivariable models. In the second phase, null models were fitted to quantify the strength of patient-level clustering within the data for each outcome variable, estimated from the intraclass correlation coefficient (ICC). Following this, a series of multivariable regressions were constructed, and backward eliminations were performed to identify which predictors were associated with each outcome. Variables were removed sequentially in order of poorest fit (highest BIC) from univariable modelling, with improvement in model fit assessed via likelihood-ratio tests (LRTs). At the end of this process we re-checked that dropped variables did not improve model fit until a final model was ascertained. Inferential statistics were obtained using the STATA statistical software, Version 15 and all analyses were performed at a level of statistical significance set at *p* < .05.

## Results

Of a possible total of 257 carers that were assessed, 254 (98.8%) carers and 198 patients with first-episode psychosis consented to publication of their data. Carer descriptives revealed that approximately two-thirds were female (67.3%), parental carers (82.3%), who lived with the patient (86.2%). Carers had a median of 40.0 hours of face-to-face contact with the patient per week.

Patient descriptives revealed that most were male (61.6%), with a primary diagnosis of Schizophrenia (71.8%) and a median illness onset of 20.9 years. The median length of psychosis was 14.8 months. The majority did not have a partner (87.3%) and were unemployed (83.5%). A full list of carer and patient demographic and clinical characteristics are summarised in Table 2.

**Table 2.** Carer and patient demographic and clinical characteristics.

Variable	*n*	Descriptive value
Carer characteristics		
Age (years): mean (*SD*), range	253	49.4 (10.9), 18.1–74.3
Gender (female), *n* (%)	254	171 (67.3)
Religion, *n* (%)	239	
No religion		19 (7.9)
Christian		109 (45.6)
Muslim		51 (21.3)
Sikh		15 (6.3)
Hindu		33 (13.8)
Other religion		12 (5.0)
Ethnicity, *n* (%)	252	
White		84 (33.3)
Asian		59 (23.4)
Black		50 (19.8)
Mixed race/other		97 (38.8)
English is carer’s first language, *n* (%)	251	150 (59.8)
Born in United Kingdom, *n* (%)	250	97 (38.2)
Age carer came to the United Kingdom (years): median (*SD*), range	149	22.0 (12.1), 0.25–55.0
Married/has a partner, *n* (%)	253	184 (72.7)
In paid employment, *n* (%)	252	156 (61.9)
Relationship to patient, *n* (%)	254	
Parent/step-parent		209 (82.3)
Sibling		15 (5.9)
Partner		21 (8.3)
Other relation		9 (3.5)
Primary carer for patient, *n* (%)	252	220 (87.3)
Caring for patient continually since the onset of psychosis, *n* (%)	253	239 (94.5)
Length of caring for patient since onset of psychosis (months): median (*SD*), range	244	14.0 (15.2), 0.50–72.0
Lives with patient, *n* (%)	254	219 (86.2)
Hours of face-to-face contact per week: median (*SD*), range	245	40.0 (33.0), 0–143.0
High face-to-face contact (⩾35 hours), *n* (%)	245	151 (61.6)
Cares for another person, *n* (%)	254	106 (41.7)
Care for another person with psychosis, *n* (%)	251	16 (6.4)
Patient characteristics		
Age (years): median (*SD*), range	198	22.5 (4.94), 14.0–36.4
Gender (female), *n* (%)	198	76 (38.4)
Diagnosis category	195	
Schizophrenia spectrum		140 (71.8)
Affective psychoses		34 (17.4)
Other diagnosis		21 (10.8)
Religion, *n* (%)	149	
No religion		23 (15.4)
Christian		56 (37.6)
Muslim		35 (23.5)
Sikh		7 (4.7)
Hindu		21 (14.1)
Other religion		7 (4.7)
Ethnicity, *n* (%)	189	
White		68 (36.0)
Asian		60 (31.7)
Black		37 (19.6)
Mixed race/other		24 (12.7)
English as first language, *n* (%)	194	161 (83.0)
Married/has a partner, *n* (%)	197	25 (12.7)
In paid employment, *n* (%)	194	32 (16.5)
Age at illness onset (years): median (*SD*), range	187	20.9 (5.03), 11.5–35.3
Duration of untreated psychosis (months): median (*SD*), range	176	1.00 (7.53), 0–51.0
Length of psychosis (months): median (*SD*), range	185	14.8 (15.4), 0.23–68.0
Inpatient at time of carer’s assessment, *n* (%)	194	20 (10.1)

*SD*: standard deviation, *n*: number of cases in analysis.

Table 3 details the final multivariable models for total negative burden and each of the eight ECI subscales. In support of our hypotheses, negative beliefs about the consequences of the illness for the patient (*p* < .001) and the carer (*p* = .002), as well as avoidant-focussed coping (*p* = .006), were independently associated with total negative burden. Avoidant-focussed coping also predicted burden by difficult behaviours (*p* < .001), stigma (*p* = .027), the effect on family (*p* = .001) and loss (*p* < .001).

**Table 3.** Final multivariable linear regression models of total negative burden and the eight ECI subscales.

Variable	B [95% CI]	*SE*	*t*	*p* value	Prob > χ²
*Model 1*: Total negative burden (*n* = 209)				Global *p* < .001	
Negative illness beliefs about the consequences for the patient (H5)	.014 [.010–.018]	.002	6.46	*p* < .001	*p* < .001
Negative illness beliefs about the consequences for the carer (H5)	.008 [.003–.013]	.003	3.19	.002	.002
Avoidant-focussed coping (H6)	.010 [.003–.016]	.003	2.79	.006	.008
Patient relationship status (in a relationship/married)	−.075 [−.149 to −.001]	.038	−2.00	.047	*p* < .001
				Adjusted *R*^²^= .4620 ICC = .512	
*Model 2*: Difficult behaviours (*n* = 184)				Global *p* < .001	
Negative illness beliefs about the consequences for the patient	.018 [.011–.025]	.004	4.98	*p* < .001	*p* < .001
Negative illness beliefs about the cyclical nature of the illness	.025 [.007–.043]	.009	2.74	.007	.008
Avoidant-focussed coping (H6)	.024 [.011–.036]	.006	3.80	*p* < .001	*p* < .001
Patient inpatient status (inpatient at the time of carer’s assessment)	.158 [.008–.307]	.076	2.08	.039	.036
Carer relationship status (in a relationship/married)	−.131 [−.227 to −.036]	.049	−2.71	.007	.008
				Adjusted *R*^²^ = .3495 ICC = .510	
*Model 3*: Negative symptoms (*n* = 197)				Global *p* < .001	
Negative illness beliefs about the consequences for the patient	.024 [.018–.030]	.003	8.23	*p* < .001	*p* < .001
Patient sex (female)	−.075 [−.154 to .005]	.040	−1.86	.065	.049
				Adjusted *R*^²^ = .2637 ICC = .574	
*Model 4*: Stigma (*n* = 193)				Global *p* < .001	
Negative illness beliefs about the consequences for the carer	.011 [.002–.020]	.005	2.33	.021	.019
Negative illness beliefs about the consequences for the patient	.010 [.002–.019]	.004	2.38	.018	.017
Avoidant-focussed coping (H6)	.015 [.002–.027]	.007	2.23	.027	.025
Patient employment status (employed)	−.189 [−.313 to −.064]	.063	−2.99	.003	.003
				Adjusted *R*^²^ = .2385 ICC = .040	
*Model 5*: Problems with services (*n* = 196)				Global *p* < .001	
Negative illness beliefs about the consequences for the carer	.010 [.005–.016]	.003	3.68	*p* < .001	*p* < .001
Carer age	.004 [.001–.008]	.002	2.59	.010	.039
Problem-focussed coping	.010 [.003–.017]	.003	2.94	.004	.007
Carer employment status (employed)	.113 [.040–.185]	.037	3.07	.002	.006
				Adjusted *R*^²^ = .1483 ICC = .206	
*Model 6*: Effect on family (*n* = 181)				Global *p* < .001	
Negative illness beliefs about the consequences for the patient	.015 [.009–.021]	.003	4.96	*p* < .001	*p* < .001
Avoidant-focussed coping (H6)	.020 [.009–.032]	.006	3.44	.001	.002
Carer age	−.004 [−.008 to −.001]	.002	−2.34	.020	.008
				Adjusted *R*^²^ = .2271 ICC = .064	
*Model 7*: Dependency (*n* = 195)				Global *p* < .001	
Negative illness beliefs about the consequences for the carer	.010 [.003–.016]	.003	2.94	.004	.003
Negative illness beliefs about the consequences for the patient	.010 [.005–.016]	.003	3.54	*p* < .001	*p* < .001
Carer face-to-face contact	.001 [.000–.002]	.000	2.71	.007	.007
Patient employment status (employed)	−.118 [−.201 to −.035]	.042	−2.81	.005	.004
				Adjusted *R*^²^ = .2989 ICC = .522	
*Model 8*: Loss (*n* = 194)				Global *p* < 0001	
Negative illness beliefs about the consequences for the patient	.012 [.007–.017]	.003	4.68	*p* < .001	.005
Avoidant-focussed coping (H6)	.021 [.012–.031]	.005	4.42	*p* < .001	*p* < .001
Patient employment status (employed)	−.104 [−.198 to −.011]	.047	−2.21	.029	.003
Emotion-focussed coping	.006 [.000–.011]	.003	1.97	.050	.040
Patient relationship status (in a relationship/married)	−.117 [−.229 to −.005]	.057	−2.07	.040	.037
				Adjusted *R*^²^ = .2976 ICC = .102	
*Model 9*: Need to backup (*n* = 198)				Global *p* < .001	
Negative illness beliefs about the consequences for the carer	.009 [.002–.015]	.003	2.51	.013	.012
Negative illness beliefs about the consequences for the patient	.009 [.003–.015]	.003	2.92	.004	.004
Patient employment status (employed)	−.102 [−.192 to −.011]	.046	−2.21	.028	.040
Emotion-focussed coping	.009 [.003–.014]	.003	3.16	.002	.030
				Adjusted *R*^²^ = .2637 ICC = .735	

*n* = number of cases in analysis; B = beta value; CI = 95% confidence interval; *SE* = standard error; *t* = *t*-test statistic; prob > χ² = Wald (LRT) statistic; H5= supports hypothesis 5; H6 = supports hypothesis 6.

### Summary of Independent Predictors

Of 43 potential predictor variables that were investigated, 14 variables were independently associated with carer burden in the multivariable models. Beliefs relating to the consequences of the illness for the patient and the carer, behavioural avoidance, and patient employment status were the variables most commonly associated with carer burden outcomes. Negative beliefs about the consequences of the illness for the patient predicted eight types of burden (total negative burden, difficult behaviours, negative symptoms, stigma, effects on family, dependency, loss and need to backup). Negative beliefs about the consequences of the illness for the carer predicted five types of carer burden (total negative burden, stigma, problems with services and dependency and need to backup), as did behavioural avoidance (total negative burden, difficult behaviours, stigma, effects on family and loss). Patient employment status predicted four types of burden (stigma, dependency, loss and a need to backup). Carer age predicted two types of burden (problems with services and effect on family), as did patient relationship status (total negative burden and loss) and emotion-focussed coping (loss and need to backup). The remaining seven variables were associated with one type of carer burden: patient inpatient status, patient sex, carer relationship status, carer employment status, carer face-to face contact, problem-focussed coping and carer beliefs in a cyclical illness.

## Discussion

### Comparison with previous literature

With regard to our sample characteristics and contrary to previous research (Addington et al., 2003; Boydell et al., 2014), we did not find any significant relationships between total carer burden and the following variables: patient age, patient diagnosis and patient illness onset age.

With regard to illness beliefs and consistent with previous research (Onwumere et al., 2008), carers who perceived greater negative consequences of the illness for both themselves and the patient, reported higher levels of burden. However, contrary to previous research (Onwumere et al., 2008), we did not find a significant relationship between carer perceptions of a longer illness timeline and burden.

In terms of coping styles, consistent with previous research (Cotton et al., 2013; Hinrichsen & Lieberman, 1999; Onwumere et al., 2011; Scazufca & Kuipers, 1999), we found that carers who employed more avoidant-focussed coping styles reported greater burden, and specifically in the domains of difficult behaviours, stigma, the effect on family and loss. Contrary to previous research (Cotton et al., 2013), we did not find a significant relationship between avoidant-focussed coping and burden by negative symptoms, problem with services, dependency or a need for backup. We also did not find a significant relationship between emotion-focussed coping and the following variables: total negative burden, burden by dependency and burden by the effect on family (cf. Möller-Leimkühler, 2005; Tennakoon et al., 2000). Furthermore, we did not find a significant relationship between problem-focussed coping and burden by the effect on family (cf. Tennakoon et al., 2000).

### Clinical and theoretical implications

Given their substantial contribution in optimising patient outcomes, recommendations to offer treatment and support to carers of individuals with psychosis is now included in the National Institute for Health and Care Excellence (2014) treatment guidelines. Yet, carers have often voiced feeling marginalised, neglected and excluded by mental health services (Cree et al., 2015; Giacco et al., 201). Careful consideration of how services might effectively address the needs of carers is evidently required and should be prioritised. We would argue for a more systematic and informed approach when supporting carers, especially considering the chronicity of psychotic disorders and the negative impact the illness may have on their own wellbeing and health status.

Our findings would suggest that carers would benefit from being screened for burden responses at the onset of patient psychosis. Identifying carers vulnerable to a conceivably preventable morbidity is crucial given that carers have been shown to access their GPs less than non-carer groups, and as a consequence, their needs may go unrecognised (McCrone et al., 2005). However, in a climate of increasing service pressures, prioritisation of carer assessments may be required. Our findings can be used to inform this process. Each of the eight sample characteristics predictive of higher carer burden in this study (carer employment status, carer relationship status, carer age, patient relationship status, patient employment status, patient sex, patient inpatient status and hours of face-to-face contact) can be viewed as independent risk factors, and prioritisation of assessments may be considered depending on number of co-existing risk factors a carer presents. Psychological interventions offered thereafter would require recognition of the carer’s ongoing and valued contribution and careful consideration of strategies to optimise both carer and patient outcomes. All carers should be provided with balanced information related to the potential challenges they may experience as part of their caregiving role, including the impact this role may have on their own health status. This may help to emphasise the importance of seeking help and engaging with services at an early stage and may also provide a gateway for clinicians to highlight and offer any suitable services that are already in place. Identifying and normalising the challenging aspects of the caregiving role may also help to counteract unhelpful or negative beliefs about their ability to cope and could possibly reduce the likelihood of inauspicious comparisons to others. Following this, carers could be encouraged to evaluate − and potentially alter − unhelpful illness beliefs and coping strategies related to their caregiving role, in favour of more adaptive responses and coping strategies. As mirrored in our results, particular attention should be paid to negative beliefs about the consequences of the illness for themselves and the patient, as well as behavioural avoidance, as these variables were most commonly associated with burden outcomes.

In conjunction, referring patients to employment and voluntary services is also strongly encouraged and should be continually emphasised by services. Indeed, previous research has shown that the onset of psychosis is frequently associated with a considerable decline in employment, especially following contact with mental health services (Rinaldi et al., 2010). This is in part due to cautious messages from mental health professionals about the risk of potential relapse (Bassett et al., 2001). Yet individuals with psychosis often identify employment as one of their main goals and view the ability to find a job or return to work as a marker of their recovery (Rinaldi et al., 2010; Secker et al., 2001). As such, employment can signify structure and purpose enabling patients to take on a stigma-free role that is associated with a positive identity (Rinaldi et al., 2010), which may, in turn, further help to improve carergiver outcomes.

### Strengths and limitations

Where our predictors are concerned, this study contains the largest and most ethnically diverse sample to date, and is the only one to assess a wide range of illness beliefs, as measured by the IPQS-RV. Our study is also one of the few to assess multiple carers per patient and to statistically control by using a multilevel analysis. However, our study is not without limitations. First, corrections for multiple testing were not applied, which may have introduced Type I errors. Second, the inherent limitations of self-reported data must be recognised as well as the absence of an independent observer. Consideration must also be given to the fact that the subsequent journey and presentation of burden may vary between carers. Finally, the cross-sectional design of the study does not allow conclusions to be drawn about the way in which burden varies over time. As caring for an individual with psychosis is a chronic stressor (Poon et al., 2017), it is important that a time perspective is included to address questions pertaining to causality.

### Future research

Future research should seek to replicate these results using different carer populations. As the percentage of variance explained by the identified predictors was relatively low in all of our multivariable models (14.83%–46.20%), research may also consider exploration of a wider range of sample characteristics (e.g. patient and carer educational attainment), or other psychological factors (e.g. carer beliefs about the causes of the illness) which might also predict carer burden. Future research should also seek to employ a longitudinal, multi-measurement point design which would be better equipped to capture the developmental trajectory and maintenance of carer burden over time. Additional inclusion of open-ended questions would allow for a more detailed and informed understanding of carer burden ontology and may also identify ways in which services can further help people who find themselves in a caregiving role.

## Conclusion

Our findings highlight a need to screen carers for burden responses at the onset of psychosis and can be used to inform prioritisation of clinical assessments by highlighting those ‘at-risk’ of experiencing burden. Unhelpful illness beliefs and coping strategies may be targeted as part of therapeutic intervention, though further research is required to confirm present findings and to assess the developmental trajectory of carer burden over time.
